# QTL Mapping of Soybean (*Glycine max*) Vine Growth Habit Trait

**DOI:** 10.3390/ijms241914770

**Published:** 2023-09-30

**Authors:** Jian Song, Kanglin Liu, Xuezhen Yang, Yijie Chen, Yajun Xiong, Qichao Yang, Jing Wang, Zhihao Zhang, Caiyu Wu, Jun Wang, Lijuan Qiu

**Affiliations:** 1 College of Life Science, Yangtze University, Jingzhou 434025, China; songjian@yangtzeu.edu.cn (J.S.); cywushuang@163.com (C.W.); 2 College of Agriculture, Yangtze University, Jingzhou 434025, China; liukanglin.st@yangtzeu.edu.cn (K.L.); yangxuezhen@haosail.com (X.Y.); 202071633@yangtzeu.edu.cn (Y.C.); 201771383@yangtzeu.edu.cn (Y.X.); 2021710693@yangtzeu.edu.cn (Q.Y.); 2021710759@yangtzeu.edu.cn (J.W.); 3 National Key Facility for Crop Gene Resources and Genetic Improvement, Institute of Crop Science, Chinese Academy of Agricultural Sciences, Beijing 100081, China; zhihao18741993@163.com

**Keywords:** *Glycine soja*, vine growth habit, QTL mapping

## Abstract

The vine growth habit (VGH) is a notable property of wild soybean plants that also holds a high degree of importance in domestication as it can preclude using these wild cultivars for breeding and improving domesticated soybeans. Here, a bulked segregant analysis (BSA) approach was employed to study the genetic etiology of the VGH in soybean plants by integrating linkage mapping and population sequencing approaches. To develop a recombinant inbred line (RIL) population, the cultivated Zhongdou41 (ZD41) soybean cultivar was bred with ZYD02787, a wild soybean accession. The VGH status of each line in the resultant population was assessed, ultimately leading to the identification of six and nine QTLs from the BSA sequencing of the F_4_ population and F_6_-F_8_ population sequence mapping, respectively. One QTL shared across these analyzed generations was detected on chromosome 19. Three other QTLs detected by BSA-seq were validated and localized to the 90.93 kb, 2.9 Mb, and 602.08 kb regions of chromosomes 6 and 13, harboring 14, 53, and 4 genes, respectively. Three consistent VGH-related QTLs located on chromosomes 2 and 19 were detected in a minimum of three environments, while an additional six loci on chromosomes 2, 10, 13, and 18 were detected in at least two environments via ICIM mapping. Of all the detected loci, five had been reported previously whereas seven represent novel QTLs. Together, these data offer new insights into the genetic basis of the VGH in soybean plants, providing a rational basis to inform the use of wild accessions in future breeding efforts.

## 1. Introduction

The domestication of soybean (*Glycine max* (L.) Merr.) plants from wild *Glycine soja* first occurred in East Asia [1,2,3]. At present, wild soybeans represent a potentially invaluable resource that may harbor elite alleles capable of broadening the genetic basis of domesticated soybean plants while improving particular traits of interest, such as high protein and biotic and abiotic stress tolerances. The vine growth habit (VGH) of these wild plants, however, hinders efforts to effectively breed them with cultivated soybean cultivars [2,4]. An essential step in the process of soybean domestication is the transition from the twining growth tendency of wild soybeans to the upright growth of widely cultivated varieties [4]. Efforts to fully understand the genetic etiological basis of the VGH will aid the effective utilization of wild resources to enable the more effective breeding-based improvement in soybean crops. As such, many research efforts have sought to clarify the genetic regulation of the VGH to provide an evidence-based foundation for molecular breeding.

The mechanisms responsible for regulating VGH are complex, as evidenced by the divergent vining growth of the offspring produced through various crosses [2,4,5,6]. The VGH is a quantitative trait influenced by many genes, varying across populations of different backgrounds and developmental stages [2,4]. For example, one study of an interspecific recombinant inbred line (RIL) identified two QTLs associated with the twining growth of mature soybean plants designated as *qTH-D1b* and *qTH-G* that were located on chromosomes 2 and 18, respectively [5]. Genotyping sequencing data derived from two *Glycine max* × *Glycine soja* populations also revealed that of 132 domestication-associated QTLs, 12 of which were associated with the growth habit, only the QTLs *qGH-19-2* (PVE = 5 and 10) were identified in both analyzed populations [6]. When studying two RIL populations from crossing the wild soybean accession PI342618B with two distinct types of cultivated soybeans, seven and five QTLs were discovered in the flowering (R1) and mature (R8) phases, respectively. These QTLs were mapped to chromosomes 1, 13, 18, and 19, and major loci included *qVGH-18-1*, *qVGH-18-2*, *qVGH-19-3*, and *qVGH-19-4* (R^2^ > 10%, detection time ≥2), although of these QTLs, only *qVGH-18-2* was consistently identified in both of these populations across cropping years and growth stages. The gibberellin oxidase (GAox) *Glyma18g06870* (*VGH1*) was identified as a candidate gene within the *qVGH-18-2* region as it exhibited significant divergence between soybean plants with vining and upright growth with an FST > 0.25 [2]. In another study, major growth-related QTLs were identified on chromosome 11 in the W05 soybean cultivar and chromosome 13 in the Wm82 cultivar, explaining 16–32% of the variance in a wild W05× cultivar C08 RIL population. The latter of these two genes was also associated with a copy number variation (CNV) in the apical bud-expressed gibberellin 2-oxidase 8A/B(GA2ox8) gene, with a positive correlation between gene copy numbers and expression levels. In contrast, these copy numbers were negatively correlated with the trailing growth and shoot length [7]. Three and one QTLs for VG were detected in the ZH39 × NY27-38 and NY36-87 population of the RILs, respectively, located on chromosomes 2, 17, and 19 [4].

For vine-type plants, shoot apical meristems (SAMs) are indeterminate such that they can continuously grow from the vegetative to the reproductive state, whereas for plants with erect or semierect growth, SAMs are determinate such that they cease growing after flowering, suggesting a potentially close genetic link between the VGH and stem growth habit. In soybean plants, two genes designated *Dt1* and *Dt2* have been found to control the stem termination type [8,9]. The *Dt1* gene is an ortholog of the Arabidopsis *TERMINAL FLOWER 11* (*TFL11*) gene [8,10,11]. It may regulate determinate growth due to an earlier drop in the expression of *GmTFL1b* coinciding with floral induction, despite functioning generally in the noninductive flowering phase [8]. *Dt2* is a gain-of-function MADS-domain factor gene capable of specifying semideterminacy, apparently via repressing *Dt1* expression in SAMs and promoting early SAM conversion into reproductive inflorescences [9,11].

The present study was developed to clarify the genetic architecture of the VGH further. To that end, we conducted bulked segregant analysis (BSA) sequencing and population resequencing of an RIL population derived from crossing the Zhongdou41 (ZD41) cultivar with the wild ZYD02787 accession. Through these approaches and associated mapping analyses, the candidate genes associated with major VGH-related QTLs were screened in different growth environments.

## 2. Results

### 2.1. VGH Characterization of Parental and RIL Soybean Plants

**Figure 1:**
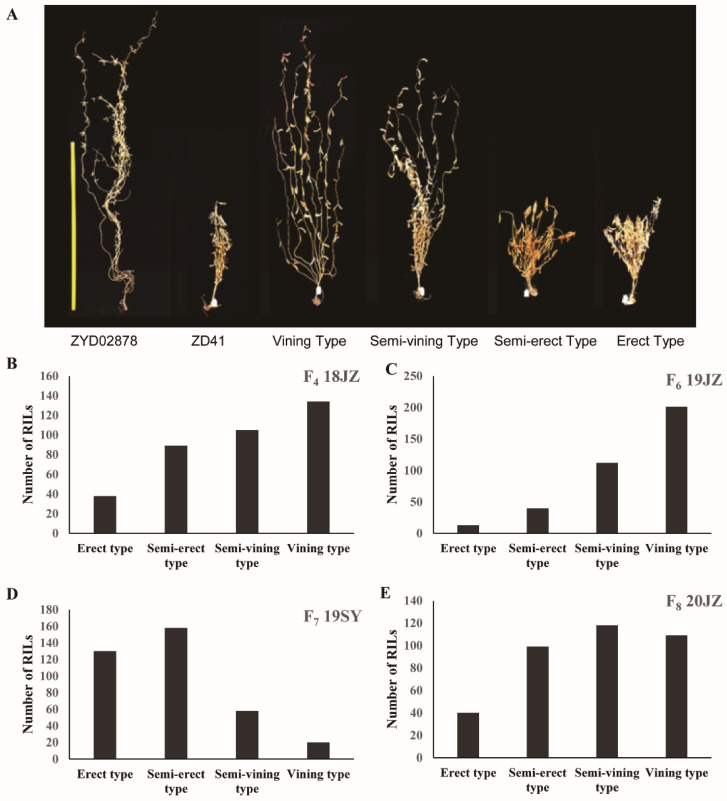
VGH phenotypic and frequency distributions for an RIL population derived from crossing ZD41 × ZYD02878 (*n* = 366). (**A**) ZD41, ZYD02878, and four VGH phenotypes; (**B**) VGH data for the F_4_ RIL population in 2018JZ; (**C**) VGH data for the F_6_ RIL population in 2019JZ; (**D**) VGH data for the F_7_ RIL population in 2019SY; and (**E**) VGH data for the F_8_ RIL population in 2020JZ.

To begin exploring the genetic regulation of the soybean VGH, an RIL population was generated by crossing the ZD41 cultivar and the wild ZYD02878 accession. Vining-type growth was evident for all members of the F_1_ generation, consistent with vining-type growth being dominant over upright growth. Four phenotypes were isolated in the F_2_ generation, including erect-, semierect-, semivining-, and vining-type growth (Figure 1A). The F_4_ RIL population was grown in Jingzhou in 2018, and the F_6_–F_8_ populations were grown in three different environments (19SY, 19JZ, and 20JZ). In the F_6_–F_8_ populations, 3.55%, 35.53%, and 10.92% of lines exhibited erect growth, respectively (Figure 1B–E). This suggests that vining-type growth is a complex trait under the control of more than two genes. Vining-type growth was more common than erect growth for all the analyzed generations, with a higher proportion of upright growth having been observed in Sanya (35.51%) relative to Jingzhou, potentially owing to the higher temperatures and lower levels of rainfall in the former region.

### 2.2. BSA-Based Identification of VGH-Associated Loci

Two bulk DNA samples from the F_4_ RIL population (vining-type and erect-type bulk samples) were used for sequencing with an Illumina instrument, yielding 60.63 Gbp of data at an average sequencing depth of 25.00x. After filtering these reads, 103,459,889 and 97,176,238 clean reads were obtained from the vining-type and erect-type mixed pools, respectively, with both pools exhibiting >97% genomic coverage and >99% genomic coverage at 1x depth, respectively. When comparing the genomic variants between these two DNA bulk pools by using GATK packages, 1,079,331 single nucleotide polymorphisms (SNPs) and 253,477 small (<50 bp) insertions/deletions (InDels) were identified.

**Figure 2:**
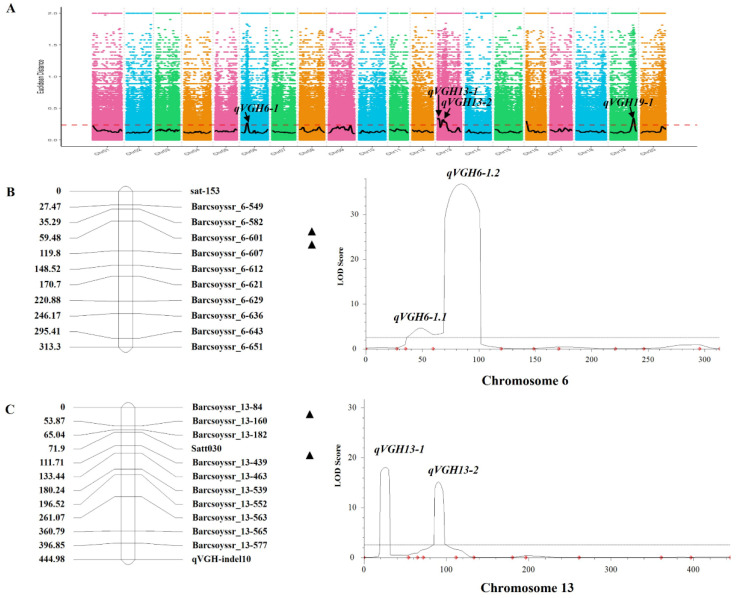
VGH-related QTL mapping in the F_4_ RIL population. (**A**) QTLs identified through BSA analyses. (**B**,**C**) ICIM-ADD mapping on Chr6 (**B**) and Chr13 (**C**). Black triangles represent QTLs, while red diamonds indicate SSR marker locations.

Based on these identified SNPs and InDels, six candidate QTLs were identified on chromosomes 6, 9, 13, 16, and 19, with all of these QTLs other than *qVGH-9-1* harboring both SNPs and InDels in these analyses (Table 1, Figure 2A). The QTL identified on chromosome 6, designated *qVGH6-1*, exhibited a physical distance of 2.86 Mb and was found to harbor 585 total genes. Two QTLs were identified on chromosome 13, including the 4.41 Mb QTL *qVGH13-1* (Chr13:0…4,410,000) carrying 314 genes and the 10.13 Mb QTL *qVGH13-2* (Chr13:8,030,000…18,160,000) harboring 740 genes. The QTL identified on chromosome 16, *qVGH16-1* (Chr16:0…1,900,000), exhibited a physical distance of 1.9 Mb and was found to have 379 genes. The QTL identified on chromosome 19, *qVGH19-1* (Chr19:42,250,000…47,850,000), spans a physical distance of 5.60 Mb and contains 1062 genes.

### 2.3. Identifying and Fine Mapping QTL in F_4_ RILs

To identify the candidate regions on chromosomes 6 and 13 identified via BSA mapping but absent in prior studies (*qVGH6-1*, *qVGH13-1*, and *qVGH13-2*), 23 SSR markers in these candidate regions that were polymorphic between the two parental lines were used for genotype identification in the F_4_ RIL population. The chromosome 6 interval contained two QTLs, of which *qVGH6-1.1* was located in a 396.80 kb region between the Barcsoyssr_6-582 and Barcsoyssr_6-601 markers (11,032,521…1,429,318) with an LOD of 4.62 and a PVE of 1.41%. In addition, *qVGH6-1.2* was in a 90.93 kb region located between the Barcsoyssr_6-601 and Barcsoyssr_6-607 markers (11,429,373…11,520,306) with an LOD of 36.83 and a PVE of 5.63% (Figure 2B). This region on chromosome 6 exhibited an ADD effect of 0.77 and was found to contain 14 genes. The QTLs on chromosome 13 were located between the Barcsoyssr_13–84 and Barcsoyssr_13–160 markers and between the satt030 and Barcsoyssr_13–439 markers. *qVGH13-1* was mapped to a 2.9 Mb region (1,641,266 to 3,015,232) containing 53 genes, with an LOD of 18.08 and a PVE of 4.88%, while *qVGH13-2* was mapped to a 602.08 kb region (8,722,749…9,324,831) containing 4 genes with an LOD of 15.14 and a PVE of 4.86% (Figure 2C).

### 2.4. VGH-Related QTL Analyses in the F_6_-F_8_ Population Derived from ZD41 × ZYD02878

**Figure 3:**
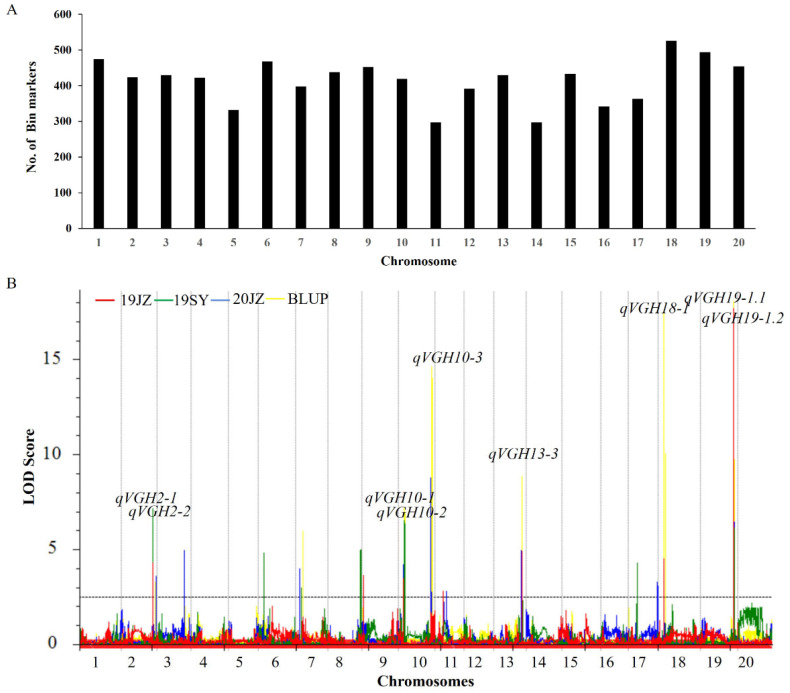
VGH-related QTL mapping in the F_6_–F_8_ RIL population. (**A**) The number of bin markers on all chromosomes. (**B**) Composite interval QTL mapping for the VGH of the 19JZ (red), 19SY (green), 20JZ (blue), and BLUP (yellow) populations by using the ICIM-ADD approach.

To validate the finding about the VGH, the phenotypic data obtained over two years, two locations, and three environments were analyzed by using ICIM-ADD mapping. To mitigate the influence of various environmental conditions on the outcomes of mapping, the calculation of the BLUP values was performed for the three test environments. These BLUP values were then utilized as phenotypic data to conduct mapping. Genotyping was performed by using 8,284 bin markers identified based on the sequencing of the RIL population (ZD41 × ZYD02878), ranging from 297 to 526 per LG, with an average genetic distance of 479.98 cM [12] (Figure 3A). These analyses identified nine VGH-related loci present in at least two environments on chromosomes 2, 10, 13, 18, and 19 (Table 2, Figure 3B). The *qVGH19-1* QTL was detected in three environments and BLUP data. It was further subdivided via ICIM mapping into the *qVGH19-1.1* and *qVGH19-1.2* QTLs, of which *qVGH19-1.1* had a higher PVE (5.73–14.51%) in three environments and in the BLUP data, while the PVE of *qVGH19-1.2* ranged from 4.59 to 5.20% in two environments and the BLUP data (Supplementary Table S1). The *qVGH19-1* QTL coincided with the BSA-seq results, and the stem-growth habit-related gene *Dt1* was located in this range. *qVGH2-1* was detected when analyzing the 19JZ, 19SY, and BLUP, while other QTLs were detected in only two environments, including *qVGH2-2*, *qVGH10-1*, *qVGH10-2*, *qVGH10-3*, *qVGH13-3*, and *qVGH18-1* (Figure 3B). Three loci were detected on chromosome 10, ranging from 200 kb to 910 kb in size (Table 3).

### 2.5. Candidate Gene Analyses

To narrow the scope of the candidate gene analyses, the QTL regions that were shared across environments and analytical methods were selected, yielding a list of 143 total candidate genes within the *qVGH6-1.2*, *qVGH13-1*, *qVGH13-2*, *qVGH13-3*, *qVGH19-1.1*, and *qVGH19-1.2* QTLs. Among these, four of the candidate genes were only present in the Wm82.a4.v1 version, whereas they were absent from the Wm82.a2.v1 version. Owing to the domestication-related nature of the VGH, *F_ST_* values were calculated for the 139 retained genes, leading to the identification of just 23 candidate genes with an *F_ST_* of >0.6 (Landraces vs. wild and improved vs. wild) in coding sequence (CDS) regions that may be subject to domestication-related selection (Supplementary Table S2).

Six candidate genes of interest were identified in the *qVGH6-1.2* region, including *Glyma.06G140300* encoding a GroES-like zinc-binding alcohol dehydrogenase family protein, *Glyma.06G140600* encoding a RING/U-box superfamily protein, *Glyma.06G140700* with an unknown function, *Glyma.06G140800* encoding a metallohydrolase/oxidoreductase superfamily protein, *Glyma.06G141100* encoding a leucine-rich repeat protein kinase family protein, and *Glyma.06G141300* with an unknown function (Table 3). These six genes harbored 11 SNPs, and four small InDels were identified in the *Glyma.06G140600*, *Glyma.06G140700*, and *Glyma.06G141100* genes (Supplementary Table S3). Gene atlas analyses indicated that *Glyma.06G141100* is coexpressed with genes in the stem-specific coexpression subnetwork with higher expression levels in the stem, suggesting that this gene may play an essential role in shaping the VGH phenotypic variability in soybean. Low *Glyma.06G140700* expression was also evident in stems (Supplementary Table S4), and *Glyma.06G140100* was found to encode a calcium-dependent lipid-binding (CaLB) domain family protein.

Three genes were selected in the *qVGH19-1.1* region, including *Glyma.19G192900*, *Glyma.19G193400*, and *Glyma.19G194600*. While *Dt1* (*Glyma.19G194300*) is also present within this interval, it was not selected. Two genes exhibited higher expression levels in stem tissues, including *Glyma.19G192800*, which encodes starch branching enzyme 2.1, and *Glyma.19G193300*, which encodes a calmodulin-binding motif family protein. The growth-regulating factor 4 gene *Glyma.19G192700* also has the potential to impact the VGH by influencing stem growth (Table 3).

Six candidate genes were additionally selected in the *qVGH19-1.2* region, including *Glyma.19G202300* encoding a VQ motif-containing protein, *Glyma.19G202800* (unknown function), *Glyma.19G203700* and *Glyma.19G203800* encoding ubiquitin-specific protease 13, *Glyma.19G204200* encoding a cleavage and polyadenylation specificity factor (CPSF) and a subunit protein, and *Glyma.19G204700* encoding a ubiquitin carboxyl-terminal hydrolase family protein. Of these genes, *Glyma.19G202300* and *Glyma.19G203800* were not expressed in stem tissues. Additionally, the sterile alpha motif domain-containing protein-coding gene *Glyma.19G203100* has the potential to impact the VGH through its effects on apical meristem growth (Table 3).

Seven genes in the QTL region located on chromosome 13 exhibited a CDS Fst of >0.6, including *Glyma.13G302800*, *Glyma.13G304000*, *Glyma.13G304500*, *Glyma.13G304700*, *Glyma.13G304800*, *Glyma.13G304900*, and *Glyma.13G305000*. Based on available expression data, *Glyma.13G008100* exhibited higher specific expression levels in stem tissues and encodes a stress-responsive A/B barrel domain protein. Based on functional analyses, *Glyma.13G302900* was found to encode a photosynthetic electron transfer C protein. Worth noting, *Glyma.13G304000* encodes a GH3 auxin-responsive promoter and may influence the VGH through effects on photosynthetic activity and GH3 auxin hormone activity (Table 3).

## 3. Discussion

While domesticated *G. max* generally exhibits upright bush-like growth, wild *G. soja* instead exhibits indeterminate vine-like growth [4]. The VGH is a typical quantitative trait strongly influenced by environmental conditions, with soybean plants growing in shaded field environments showing slender lodging stems, for example [13]. To identify the QTLs associated with the soybean VGH, RIL populations prepared via crossing *G. max × G. soja* were analyzed, ultimately leading to the identification of major QTLs that were conserved across environments and analytical methods.

BSA-seq and population resequencing strategies have increasingly been applied in recent years to aid in rapidly identifying the QTLs associated with a range of soybean traits of interest [12,14,15,16,17,18,19]. Here, BSA-seq and RIL population resequencing strategies identified VGH-related regions on chromosomes 2, 6, 10, 13, 18, and 19. Of these loci, *qVGH19-1* was detected across multiple environments by using both analytical approaches, and it corresponds to a stable QTL that has repeatedly been mapped in prior reports. In one past study, the growth-habit-related *qGH*-*19*-*2* locus was mapped to the same position, with respective PVE values of 10% and 5% in WP468 and WP479 [6]. Liu et al. [5] and Lu et al. [4] conducted analyses of the VGH based on the number of times the main soybean stem wrapped around a support, leading to the identification of the plant-height-related *qVG-19* and *qVG-19.1* QTLs on chromosome 9 (LG L) in two RIL populations [4]. Three QTLs associated with the VGH at maturity were detected on chromosome 19, including a 44–47 Mb region (*qVGH19-1*) that was separated into two QTLs, with *qVGH-19-3* and *qVGH-19-4* mapping to similar or identical positions to *qVGH-19-1.1* and *qVGH-19-1.2* on this chromosome [2]. The previously characterized stem-growth-related *Dt1* gene was located in *qVGH-19-1.1*, and there are phenotypic similarities between vine growth and indeterminate stem growth. However, it remains uncertain whether *Dt1* influences the VGH as the W82 female parents used in the population for *qGH-19-2* and *qVGH-19-3* exhibited the same haplotype as the wild male parents [2,6,8]. In this study, *Glyma.19G194300* harbored a nonsynonymous SNP (45184804, C-A), while *Glyma.19G192700* located within this region was found to encode growth-regulating factor 4 that may be related to the VGH.

The *qVGH2-1* QTL was detected in the analyzed populations from at least two environments in this study. Liu et al. previously reported the *qTH-D1b* QTL at *satt546*, which exhibited respective PVE values of 20.5% and 10.1% for the testing performed in 2004 and 2005 [5]. The growth-habit-related QTL *qGH-2* has also been mapped to a 44.6 Mb region of chromosome 2 in the WP479 cultivar, exhibiting a PVE value of 10.1% [6]. In a population derived from crossing ZH39 and NY27-38, *qVG-2* was mapped to a 43.3–44.3 Mb region with a PVE of 5.80–9.84% [4]. *qVGH13-3* was localized to a 39.0–39.5 Mb region near the QTL found in the R1 stage in the population NJRINP, which has been mapped to a 37.8–38.0 Mb region of the Wm82 genome [2]. Increases in the GA2ox8 gene copy number have also been reported to decrease trailing growth [7].

VGH studies are relatively rare due to the strong environmental influence and difficulties associated with phenotypic identification. Similar “vining score” rating systems have previously been reported when discussing the VGH of plants derived from two *G. max* × *G. soja* crosses [20]. In several reports, authors have classified these plants as exhibiting erect growth (upright growth for the entirety of the plant stem), semierect (trailing of the top of the main stem), semitrailing (trailing of >50% of the main stem), or trailing growth (the entirety of the main stem was wound around supporting stakes or twine) [2,7]. In other scores, vining tendencies were quantified on a numerical scale from one (indeterminate growth) to five (prolific vining growth similar to that of *G. soja*) [6]. There have even been efforts to study the VGH based on quantifying the number of times a main stem wound around its support [4,5]. These methods have enabled the identification of shared loci including *qTH-L* [5], *qGH-19-2* [6], *qVGH-19-3* [2], and *qVG-19* [4] on chromosome 19, as well as *qTH-G* [5], *qGH-18* [6], and *qVGH-18-2* [2] on chromosome 18. Here, phenotypic identification was performed by using four classical types of phenotypic classification. To enhance the precision of the finding, the loci were examined throughout four generations and in various environmental conditions. This comprehensive analysis led to the identification of nine loci that were observed in at least two environments. Notably, five of these loci (*qVGH2-1*, *qVGH13-3*, *qVGH18-1*, *qVGH19-1.1*, and *qVGH19-1.2*) exhibited significant similarity, either exact or nearly exact to previously reported QTLs. This outcome underscores the efficacy and reliability of the experimental methodology employed in this study. At the same time, our interval is relatively small compared with previous reports, which is more conducive to gene cloning. For example, in our study, *qVGH2-1* is only 200 kb, and the nine QTL intervals are all in the range of 170 kb~960 kb.

The VGH is a domestication-associated trait that developed through anthropogenic selection as it has important implications for the agricultural cultivation of soybean plants [1,3]. To gain greater insight into the genetic basis for the domestication of wild soybean plants, the genes and loci associated with domestication-related traits must be identified so that these wild resources can be more effectively utilized. *F_ST_* values are used in population genetics to evaluate the evolution of genetic variation within and among populations [21,22]. Here, *Fst* analyses of candidate genes of interest were performed based on data derived from 2214 soybean accessions. This led to the selection of 23 genes with CDS SNPs, revealing no significant domestication of the *qVGH19-1* region in line with prior reports [2]. Selection for a GH3 auxin-responsive promoter was also observed. Gibberellin (GA) and other hormones play a central role in regulating plant characteristics, and a *GmDW1* mutant has been reported to exhibit lower GA levels associated with a dwarf phenotype [23]. GA2ox8 can reportedly influence both trailing growth and shoot length [7]. *GmIAA27* codes for an AUX/IAA protein associated with dwarfing and multibranched development, relying on auxin for interactions with TIR1 and the induction of specific GH3 genes [24]. Brassinosteroids can regulate the leaf angle, and brassinolide-synthesis-related gene upregulation in maize, wheat, and rice has been reported to promote increased leaf inclination. In contrast, the downregulation of these genes has the opposite effect [25,26,27,28,29]. The GWAS results for these 2214 soybean accessions can enable VGH analyses, gene cloning, and the evaluation of soybean cyst nematode resistance.

In the present study, we combined BSA-seq, linkage mapping, and a population sequence and identified six and nine QTLs from the F_4_ BSA sequence and population sequence mapping in F_6_-F_8_, respectively. A common QTL shared by all generations was located on chromosome 19. Three additional QTLs identified (*qVGH6-1.2*, *qVGH13-1*, *and qVGH13-2*) by BSA-seq were also validated and narrowed to 90.93 kb, 2.9 Mb, and 602.08 kb on chromosomes 6 and 13, containing 14, 53, and 4 genes, respectively. Three QTLs, namely *qVGH2-1*, *qVGH19-1.1*, and *qVGH19-1.2*, were identified in a minimum of two environments and were found to be associated with the BLUP for the variable of interest VGH. These QTLs were located on chromosomes 2 and 19. Six other loci were detected in two environments or one environment and BLUP. Among them, five loci were identified in previous studies, as well as seven novel QTLs and the fine mapping of these QTLs needed for cloning the underlying genes, which will broaden our understanding of the genetics of the VGH and thus facilitate the utilization of wild resources in breeding.

## 4. Materials and Methods

### 4.1. Plant Materials and Phenotypic Analyses

The ZD41 soybean cultivar and the wild ZYD02878 soybean accession were obtained from the Chinese Academy of Agricultural Sciences. ZD41 exhibits upright growth without twining and is widely grown throughout Central China, whereas ZYD02878 is a wild variety with typical vining phenotypes originally harvested from Shanxi Province in China. ZD41 (the female parent) and ZYD02878 (the male parent) were crossed initially in 2015, and RIL populations from F_2_ to F_8_ were generated through single-seed descent, ultimately yielding a population comprising 364 lines.

All the experimental lines were cultivated in experimental stations in Sanya and Jingzhou for phenotypic analyses. The RIL-F_4_, RIL-F_6_, and RIL-F_8_ populations were planted in the experimental field of Crop Science of Yangtze University (30.37° N, 112.06° E) with a plant spacing of 30, 50, and 100 cm during the 2018, 2019, and 2020 seasons, respectively, while the RIL-F_7_ population was planted at the Sanya NanFan Research Center of CAAS (18.25° N, 109.51° E) from November to February 2020 with a plant spacing of 30 cm. The F_6_–F_8_ populations were, respectively, designated 19JZ, 19SY, and 20JZ for subsequent analyses. 19JZ, 19-SY, and 20JZ, were set up with 1, 3, and 3 replicates, respectively. In order to prevent the soybean plants from intertwining with each other, bamboo poles are erected next to each plant in the early vegetative growth stage of different generations, promoting their growth along the bamboo poles and the harvesting of individual plants. Field management adopts conventional management, which is consistent with local soybean-field production management.

The VGH phenotypic analyses were performed in the drum stage of growth, classifying plants into four categories [2,7]: Type 1 (erect type) plants with upright growth for >80% of the main stem, as in the prototypical female ZD41 parental variety; Type 2 (semierect type) plants exhibiting vertical growth for over 60% of the main stem, with slight thinning and waviness for the upper portion of the main stem but without winding; Type 3 (semivining type) plants with upright growth for over 20% of the main stem but with the middle and upper portions consisting of an elongated climbing vine with some winding; or Type 4 (vining type) plants with a weak stem that cannot stand upright, instead relying on the trunk or other objects for spreading with intense entanglement, as in the case of the paternal wild ZYD02878 accession. The chisq.test.4 function was used to perform chi-square analyses. The R reshape2, lmerTest, lme4, Reshape2, and lsmeans packages were used to compute the BLUP values, integrating samples repeatedly detected across multiple environments and multiple years while removing outlier phenotypic data to control for environmental effects on downstream data analyses.

### 4.2. Genotyping of Individual and DNA Bulk Samples

The cetyltrimethylammonium bromide (CTAB) method was used to extract whole genomic DNA from healthy leaves [30]. In order to obtain vining and erect bulk DNA samples (VB and EB, respectively), a total of 200 ng of DNA was combined from 20 vining or 20 erect F_4_ lines. These pooled DNA samples were then sequenced with an Illumina HiSeq4000 instrument based on standard recommendations for DNA sequencing. The resultant reads were cleaned and mapped to the *Glycine max* Wm82.a2.v1 reference genome from Phytozome by using a Burrows–Wheeler Aligner (BWA) with the default parameters [31]. The calling of SNPs and InDels (<50 bp) was performed by using the GATK (Genome Analysis Toolkit, v 4.2) [32]. Trait-associated regions were evaluated with a Euclidean Distance (ED) algorithm, with ED values being calculated based on the genotype and depth of different mixed pools [33].

The total genomic DNA of each F_7_ RIL was isolated, and 366 libraries were constructed via the NEB Next ^®^Ultra™ DNA Library Prep Kit for Illumina (NEB, Ipswich, MA, USA). An Illumina instrument was used for the 150 bp paired-end read-based sequencing of the DNA libraries. Clean reads were generated by filtering raw data in the FASTQ format, after which the BWA software was used to map these data to the reference genome (Wm82.a4.v1) under the default parameters [31]. SAMtools was used to call SNPs/InDels with the following settings: mpileup -m 2, -F 0.002, and -d 1000. Only the SNPs with a variable position depth of >4 and a mapping quality of >20 were retained for analysis.

### 4.3. Linkage Map Construction and QTL Mapping

After removing the anomalous SNPs following SNP calling, polymorphic SNPs were used to estimate recombinant points defined based on a difference in a bin of 15 continuous SNPs relative to another individual RIL [34]. Bin markers exhibiting identical genotypes within a 100 kb window were merged into a single bin marker, ultimately selecting 8284 bin markers across 20 chromosomes [12]. VGH-related QTLs were then identified via complete interval mapping (ICIM-ADD) by using QTL IciMapping4.0 [35] with the threshold line LOD 2.5.

### 4.4. Candidate Genes Prioritization

The Phytozome database gene models within the QTL regions were searched on Phytozome (http://www.phytozome.com, accessed on 6 March 2023). Further candidate gene screening was then performed by calculating fixation index (Fst) values based on published genomic sequencing data from 2214 soybean plants [36] by using vcftools (v0.1.13) [37], with a 100 bp window size. A coding region Fst of ≥0.6 was considered indicative of a possible domestication-associated gene [38]. Spatiotemporal analyses of candidate gene expressions were then performed by using W82 transcriptomic data (http://www.phytozome.com, accessed on 20 March 2023), yielding results in the fragments per kilobase of the transcript per million reads mapped (FPKM) format (Supplement Table S4). A candidate gene functional annotation was performed with Phytozome and the SoyBase database (http://www.soybase.org, accessed on 12 April 2023).

## Figures and Tables

**Table 1:** ED association analysis of SNPs and InDels for VGH.

QTLs	Chr	Start (W82a2)	End (W82a2)	Position (Mb)	Gene_Number	Genotype
*qVGH6-1*	6	9,580,000	12,440,000	2.86	585	SNP, InDel
*qVGH9-1*	9	41,910,000	42,920,000	1.01	187	SNP
*qVGH13-1*	13	0	4,410,000	4.41	314	SNP, InDel
*qVGH13-2*	13	8,030,000	18,160,000	10.13	740	SNP, InDel
*qVGH16-1*	16	0	1,900,000	1.90	379	SNP, InDel
*qVGH19-1*	19	42,250,000	47,850,000	5.60	1062	SNP, InDel

**Table 2:** Colocalized VGH-related QTLs were identified by using the ICIM for populations grown in three environments and BLUP.

QTL Name	Environment	Chr.	Start (W82a4)	End (W82a4)	Range (Mb)
*qVGH2-1*	19JZ,19SY,BLUP	2	44,549,340	44,748,366	0.2
*qVGH2-2*	20JZ,BLUP	2	48,133,595	49,091,931	0.96
*qVGH10-1*	19JZ,20JZ	10	7,448,517	7,648,577	0.2
*qVGH10-2*	19SY,BLUP	10	8,298,686	9,212,033	0.91
*qVGH10-3*	19SY,BLUP	10	45,339,503	45,848,959	0.51
*qVGH13-3*	19JZ,BLUP	13	39,347,224	39,548,455	0.21
*qVGH18-1*	19JZ,BLUP	18	7,843,488	8,011,289	0.17
*qVGH19-1.1*	19JZ,19SY,20JZ,BLUP	19	45,447,240	45,839,781	0.39
*qVGH19-1.2*	19SY,20JZ,BLUP	19	46,449,908	46,641,266	0.19

**Table 3:** Functional annotation of candidate genes.

QTL Name	Gene	Annotation	Fst > 0.6
*qVGH6-1.2*	*Glyma.06G141100*	Leucine-rich repeat protein kinase family protein	Yes
	*Glyma.06G140600*	RING/U-box superfamily protein	Yes
	*Glyma.06G140700*	--	Yes
*qVGH13-1*	*Glyma.13G008100*	Stress-responsive A/B Barrel Domain	No
	*Glyma.13G006500*	NUDT2, nudix hydrolase homolog 2	No
*qVGH13-2*	*Glyma.13G029500*	UDP-glucosyl transferase 85A2	No
*qVGH13-3*	*Glyma.13G302800*	Major facilitator superfamily protein	Yes
	*Glyma.13G302900*	Photosynthetic electron transfer C	No
	*Glyma.13G304000*	GH3 auxin-responsive promoter (GH3)	Yes
*qVGH19-1.1*	*Glyma.19G192700*	Growth-regulating factor 4	No
	*Glyma.19G194300*	TFL1, PEBP (phosphatidylethanolamine-binding protein) Family protein	No
*qVGH19-1.2*	*Glyma.19G203100*	Sterile alpha motif (SAM) domain-containing protein	No

## Data Availability

The data generated and analyzed during the current study are available from the corresponding author upon reasonable request.

## Supplementary Materials

The following supporting information can be downloaded at: https://www.mdpi.com/article/10.3390/ijms241914770/s1.

## References

[1] Cao D., Takeshima R., Zhao C., Liu B., Jun A., Kong F. (2017). Molecular mechanisms of flowering under long days and stem growth habit in soybean. J. Exp. Bot..

[2] Wang R., Liu L., Kong J., Xu Z., Akhter Bhat J., Zhao T. (2019). QTL architecture of vine growth habit and gibberellin oxidase gene diversity in wild soybean (*Glycine soja*). Sci. Rep..

[3] Sun S., Wang Y., Wei H., Hufnagel D.E., Wang Y., Guo S., Li Y., Wang L., Qiu L.-J. (2023). The prevalence of deleterious mutations during the domestication and improvement of soybean. Crop J..

[4] Lu Y., Zhang J., Guo X., Chen J., Chang R., Guan R., Qiu L. (2022). Identification of genomic regions associated with vine growth and plant height of soybean. Int. J. Mol. Sci..

[5] Liu B., Fujita T., Yan Z.H., Sakamoto S., Xu D., Abe J. (2007). QTL mapping of domestication-related traits in soybean (*Glycine max*). Ann. Bot..

[6] Swarm S.A., Sun L., Wang X., Wang W., Brown P.J., Ma J., Nelson R.L. (2019). Genetic dissection of domestication-related traits in soybean through genotyping-by-sequencing of two interspecific mapping populations. Theor. Appl. Genet..

[7] Wang X., Li M.W., Wong F.L., Luk C.Y., Chung C.Y., Yung W.S., Wang Z., Xie M., Song S., Chung G. (2021). Increased copy number of gibberellin 2-oxidase 8 genes reduced trailing growth and shoot length during soybean domestication. Plant J..

[8] Tian Z., Wang X., Lee R., Li Y., Specht J.E., Nelson R.L., McClean P.E., Qiu L., Ma J. (2010). Artificial selection for determinate growth habit in soybean. Proc. Natl. Acad. Sci. USA.

[9] Ping J., Liu Y., Sun L., Zhao M., Li Y., She M., Sui Y., Lin F., Liu X., Tang Z. (2014). Dt2 is a gain-of-function MADS-domain factor gene that specifies semideterminacy in soybean. Plant Cell.

[10] Liu B., Watanabe S., Uchiyama T., Kong F., Kanazawa A., Xia Z., Nagamatsu A., Arai M., Yamada T., Kitamura K. (2010). The soybean stem growth habit gene Dt1 is an ortholog of Arabidopsis TERMINAL FLOWER1. Plant Physiol..

[11] Liu Y., Zhang D., Ping J., Li S., Chen Z., Ma J. (2016). Innovation of a Regulatory Mechanism Modulating Semi-determinate Stem Growth through Artificial Selection in Soybean. PLoS Genet..

[12] Chen Y., Xiong Y., Hong H., Li G., Gao J., Guo Q., Sun R., Ren H., Zhang F., Wang J. (2023). Genetic dissection of and genomic selection for seed weight, pod length, and pod width in soybean. Crop J..

[13] Liu W., Zou J., Zhang J., Yang F., Yang W. (2015). Evaluation of Soybean (*Glycine max*) Stem Vining in Maize-Soybean Relay Strip Intercropping System. Proc. Jpn. Acad. Ser. A Math. Sci..

[14] Campbell B.W., Hofstad A.N., Sreekanta S., Fu F., Kono T.J.Y., O’Rourke J.A., Vance C.P., Muehlbauer G.J., Stupar R.M. (2016). Fast neutron-induced structural rearrangements at a soybean NAP1 locus result in gnarled trichomes. Theor. Appl. Genet..

[15] Song J., Li Z., Liu Z., Guo Y., Qiu L.J. (2017). Next-generation sequencing from bulked-segregant analysis accelerates the simultaneous identification of two qualitative genes in soybean. Front. Plant Sci..

[16] Amin G.M.A. (2019). Characterization and rapid gene-mapping of leaf lesion mimic phenotype of spl-1 mutant in soybean (*Glycine max* (L.) Merr.). Int. J. Mol. Sci..

[17] Li R., Jiang H., Zhang Z., Zhao Y., Chen Q. (2019). Combined linkage mapping and BSA to identify QTL and candidate genes for plant height and the number of nodes on the main stem in soybean. Int. J. Mol. Sci..

[18] Liu X.T., Wu X.Y., Wu W.P., Wu M., Chen J.Q., Wang B. (2021). A bean common mosaic virus-resistance gene in the soybean variant V94-5152 was mapped to the Rsv4 locus conferring resistance to soybean mosaic virus. Theor. Appl. Genet..

[19] Silva M.P., Zaccaron A.Z., Bluhm B.H., Rupe J.C., Wood L., Mozzoni L.A., Mason R.E., Yingling S., Pereira A. (2020). Bulked segregant analysis using next-generation sequencing for identification of genetic loci for charcoal rot resistance in soybean. Physiol. Mol. Plant Pathol..

[20] Carpenter J.A., Fehr W.R. (1986). Genetic variability for desirable agronomic traits in populations containing Glycine soja germplasm. Crop Sci..

[21] Holsinger K.E., Weir B.S. (2009). Genetics in geographically structured populations: Defining, estimating and interpreting *Fst*. Nat. Rev. Genet..

[22] Bhatia G., Patterson N., Sankararaman S., Price A.L. (2013). Estimating and interpreting FST: The impact of rare variants. Genome Res..

[23] Li Z.F., Guo Y., Ou L., Hong H., Wang J., Liu Z.X., Guo B., Zhang L., Qiu L. (2018). Identification of the dwarf gene GmDW1 in soybean ( *Glycine max* L.) by combining mapping-by-sequencing and linkage analysis. Theor. Appl. Genet..

[24] Su B., Wu H., Guo Y., Gao H., Wei Z., Zhao Y., Qiu L. (2022). GmIAA27 encodes an AUX/IAA protein involved in dwarfing and multi-branching in soybean. Int. J. Mol. Sci..

[25] Nakamura A. (2006). The Role of OsBRI1 and Its Homologous Genes, OsBRL1 and OsBRL3, in Rice. Plant Physiol..

[26] Hu X., Qian Q., Xu T., Zhang Y., Dong G., Gao T., Xie Q., Xue Y. (2013). The U-box E3 ubiquitin ligase TUD1 functions with a heterotrimeric G alpha subunit to regulate Brassinosteroid-mediated growth in rice. PLoS Genet..

[27] Tian X., Li X., Zhou W., Ren Y., Wang Z., Liu Z., Tang J., Tong H., Fang J., Bu Q. (2017). Transcription factor OsWRKY53 positively regulates brassinosteroid signaling and plant architecture. Plant Physiol..

[28] Liu K., Cao J., Yu K., Liu X., Gao Y., Chen Q., Zhang W., Peng H., Du J., Xin M. (2019). Wheat TaSPL8 Modulates Leaf Angle Through Auxin and Brassinosteroid Signaling1[OPEN]. Plant Physiol..

[29] Tian J.G., Wang C.L., Xia J.L., Wu L.S., Xu G.H., Wu W.H., Li D., Qin W.C., Han X., Chen Q.Y. (2019). Teosinte ligule allele narrows plant architecture and enhances high-density maize yields. Science.

[30] Doyle J. (1987). A rapid DNA isolation procedure for small quantities of fresh leaf tissue. Phytochem. Bull..

[31] Langmead B., Salzberg S.L. (2012). Fast gapped-read alignment with Bowtie 2. Nat. Methods.

[32] Mckenna A., Hanna M., Banks E., Sivachenko A., Cibulskis K., Kernytsky A., Garimella K., Altshuler D., Gabriel S., Daly M. (2010). The genome analysis toolkit: A map reduce framework for analyzing next-generation DNA sequencing data. Genome Res..

[33] Hill J.T., Demarest B.L., Bisgrove B.W., Gorsi B., Yost H.J. (2013). MMAPPR: Mutation mapping analysis pipeline for pooled RNA-seq. Genome Res..

[34] Huang X., Feng Q., Qian Q., Zhao Q., Wang L., Wang A., Guan J., Fan D., Weng Q., Huang T. (2009). High-throughput genotyping by whole-genome resequencing. Genome Res..

[35] Meng L., Li H., Zhang L., Wang J. (2015). QTL IciMapping:Integrated software for genetic linkage map construction and quantitative trait locus mapping in biparental populations. Crop J..

[36] Li Y.H., Qin C., Wang L., Jiao C., Hong H., Tian Y., Li Y., Xing G., Wang J., Gu Y. (2023). Genome-wide signatures of the geographic expansion and breeding of soybean. Sci. China Life Sci..

[37] Danecek P., Auton A., Abecasis G., Albers C.A., Banks E., Depristo M.A., Handsaker R.E., Lunter G., Marth G.T., Sherry S.T. (2011). The variant call format and VCFtools. Bioinformatics.

[38] Song Q., Hyten D.L., Jia G., Quigley C.V., Fickus E.W., Nelson R.L., Cregan P.B. (2013). Development and Evaluation of SoySNP50K, a High-Density Genotyping Array for Soybean. PLoS ONE.

