# Supplementary material for: QTL Mapping of Soybean (Glycine max) Vine Growth Habit Trait
Source: Int J Mol Sci. 2023 Sep 30;24(19):14770. doi: 10.3390/ijms241914770 (PMC10572949; doi:10.3390/ijms241914770)
Supplement: Supplementary file 1 [file ijms-24-14770-s001.zip › ijms-2553500-supplementary.pdf]

Stable1 The QTLs for VGH in 3 environments and BULP by ICIM mapping

TraitID	Chromosome	Position	LeftMarker	RightMarker	LOD	PVE(%)	Add	LeftCI(Mb)	RightCI(Mb)
19JZ	2	445.5	bin840	bin841	4.29	3.23	-0.15	44.5	44.7
19JZ	9	28.5	bin3416	bin3417	3.61	2.71	0.15	2.8	3.0
19JZ	10	74.5	bin3912	bin3913	3.46	2.59	0.14	7.4	7.5
19JZ	11	108.5	bin4367	bin4368	2.79	2.08	0.13	10.8	10.9
19JZ	13	394	bin5320	bin5321	4.90	4.09	-0.17	39.3	39.5
19JZ	18	78.5	bin6892	bin6893	4.48	3.38	-0.16	7.8	7.9
19JZ	19	456.5	bin7777	bin7778	17.66	14.51	-0.32	45.6	45.7
19SY	2	445.5	bin840	bin841	7.16	6.11	-0.20	44.5	44.6
19SY	6	77.5	bin2160	bin2161	4.82	4.04	0.17	7.6	7.8
19SY	7	82.5	bin2633	bin2634	2.97	2.47	-0.13	8.2	8.3
19SY	8	458.5	bin3374	bin3375	5.00	4.20	0.17	45.7	45.9
19SY	10	87.5	bin3921	bin3922	6.53	5.58	0.20	8.3	9.2
19SY	17	118.5	bin6569	bin6570	4.26	3.57	0.16	11.8	11.9
19SY	19	454.5	bin7775	bin7776	10.15	8.83	-0.25	45.4	45.5
19SY	19	464.5	bin7785	bin7786	6.13	5.20	-0.19	46.4	46.6
20JZ	2	489.5	bin884	bin885	3.57	3.29	-0.14	48.9	49.0
20JZ	3	372.5	bin1231	bin1232	4.94	4.59	-0.17	37.2	37.3
20JZ	7	64.5	bin2615	bin2616	3.99	3.69	-0.15	6.4	6.5
20JZ	10	74.5	bin3912	bin3913	4.21	3.90	0.16	7.4	7.5
20JZ	10	456.5	bin4200	bin4201	8.77	8.36	-0.23	45.6	45.7
20JZ	11	152.5	bin4396	bin4397	2.80	2.48	-0.12	15.2	15.3
20JZ	13	378	bin5304	bin5305	4.92	4.95	-0.17	37.7	37.9
20JZ	17	404.5	bin6801	bin6802	3.26	3.00	0.14	40.4	40.6
20JZ	19	454.5	bin7775	bin7776	5.73	5.36	-0.18	45.4	45.6
20JZ	19	464.5	bin7785	bin7786	6.42	6.03	-0.20	46.4	46.5
BLUP	2	445.5	bin840	bin841	5.94	2.74	-0.11	44.5	44.6
BLUP	2	481.5	bin876	bin877	3.28	1.48	-0.08	48.1	48.3
BLUP	7	106.5	bin2657	bin2658	5.96	2.75	-0.11	10.6	10.8
BLUP	10	87.5	bin3921	bin3922	7.19	3.36	0.12	8.7	9.3
BLUP	10	453.5	bin4197	bin4198	7.31	3.31	-0.12	45.3	45.4
BLUP	10	466.5	bin4210	bin4211	14.61	7.12	-0.17	46.6	46.7
BLUP	10	470.5	bin4214	bin4215	14.01	6.80	0.17	47.0	47.2
BLUP	13	394	bin5320	bin5321	8.86	4.84	-0.14	39.3	39.5
BLUP	18	78.5	bin6892	bin6893	17.50	8.69	-0.19	7.8	7.9
BLUP	18	96.5	bin6908	bin6909	10.04	4.75	0.15	9.6	9.8
BLUP	19	454.5	bin7775	bin7776	18.06	9.01	-0.20	45.4	45.5
BLUP	19	464.5	bin7785	bin7786	9.73	4.59	-0.14	46.4	46.5

Stable 2 The CDS Fst>0.6 genes in major QTL

Chr	Position	Fst	type	color	gene	geneType
6	11443806	0.845325	Improved vs Wild	green	Glyma.06G140300.1	CDS
6	11442295	0.802646	Improved vs Wild	green	Glyma.06G140300.1	CDS
6	11474797	0.604828	Improved vs Wild	green	Glyma.06G140600.1	CDS
6	11470251	0.872857	Improved vs Wild	green	Glyma.06G140700.1	CDS
6	11470251	0.587585	Landrace vs Wild	red	Glyma.06G140700.1	CDS
6	11481219	0.640317	Improved vs Wild	green	Glyma.06G140800.1	CDS
6	11496362	0.805488	Improved vs Wild	green	Glyma.06G141100.1	CDS
6	11496607	0.705407	Improved vs Wild	green	Glyma.06G141100.1	CDS
6	11497236	0.678474	Improved vs Wild	green	Glyma.06G141100.1	CDS
6	11496362	0.607148	Landrace vs Wild	red	Glyma.06G141100.1	CDS
6	11522083	0.843958	Improved vs Wild	green	Glyma.06G141300.1	CDS
6	11522684	0.834541	Improved vs Wild	green	Glyma.06G141300.1	CDS
6	11522083	0.713255	Landrace vs Wild	red	Glyma.06G141300.1	CDS
6	11522690	0.70231	Improved vs Wild	green	Glyma.06G141300.1	CDS
6	11522684	0.699612	Landrace vs Wild	red	Glyma.06G141300.1	CDS
13	39977464	0.680176	Improved vs Wild	green	Glyma.13G302800.1	CDS
13	39977932	0.679271	Improved vs Wild	green	Glyma.13G302800.1	CDS
13	39977678	0.675691	Improved vs Wild	green	Glyma.13G302800.1	CDS
13	39977928	0.672709	Improved vs Wild	green	Glyma.13G302800.1	CDS
13	40113409	0.604441	Improved vs Wild	green	Glyma.13G304000.1	CDS
13	40142515	0.81075	Improved vs Wild	green	Glyma.13G304500.1	CDS
13	40141801	0.789565	Improved vs Wild	green	Glyma.13G304500.1	CDS
13	40161491	0.672788	Improved vs Wild	green	Glyma.13G304700.1	CDS
13	40162007	0.66588	Improved vs Wild	green	Glyma.13G304800.1	CDS
13	40161837	0.65291	Improved vs Wild	green	Glyma.13G304800.1	CDS
13	40161898	0.643951	Improved vs Wild	green	Glyma.13G304800.1	CDS
13	40161868	0.634435	Improved vs Wild	green	Glyma.13G304800.1	CDS
13	40161862	0.630431	Improved vs Wild	green	Glyma.13G304800.1	CDS
13	40166783	0.759249	Improved vs Wild	green	Glyma.13G304900.1	CDS
13	40187515	0.782835	Improved vs Wild	green	Glyma.13G305000.1	CDS
13	40181569	0.776905	Improved vs Wild	green	Glyma.13G305000.1	CDS
13	40180821	0.772042	Improved vs Wild	green	Glyma.13G305000.1	CDS
13	40183385	0.770271	Improved vs Wild	green	Glyma.13G305000.1	CDS
13	40176835	0.761564	Improved vs Wild	green	Glyma.13G305000.1	CDS
13	40178572	0.758122	Improved vs Wild	green	Glyma.13G305000.1	CDS
13	40177383	0.750156	Improved vs Wild	green	Glyma.13G305000.1	CDS
13	40177585	0.747363	Improved vs Wild	green	Glyma.13G305000.1	CDS
19	45077823	0.640885	Landrace vs Wild	red	Glyma.19G192900.1	CDS
19	45115082	0.697218	Landrace vs Wild	red	Glyma.19G193400.1	CDS
19	45115082	0.637407	Improved vs Wild	green	Glyma.19G193400.1	CDS
19	45208914	0.680157	Landrace vs Wild	red	Glyma.19G194600.1	CDS
19	45208914	0.64908	Improved vs Wild	green	Glyma.19G194600.1	CDS
19	45923578	0.877593	Improved vs Wild	green	Glyma.19G202300.1	CDS

19	45923578	0.869822	Landrace vs Wild	red	Glyma.19G202300.1	CDS
19	45951928	0.73231	Landrace vs Wild	red	Glyma.19G202800.1	CDS
19	45951928	0.710076	Improved vs Wild	green	Glyma.19G202800.1	CDS
19	46011961	0.65206	Landrace vs Wild	red	Glyma.19G203700.1	CDS
19	46011961	0.64313	Improved vs Wild	green	Glyma.19G203700.1	CDS
19	46015763	0.715995	Landrace vs Wild	red	Glyma.19G203800.1	CDS
19	46015763	0.697255	Improved vs Wild	green	Glyma.19G203800.1	CDS
19	46039187	0.764734	Improved vs Wild	green	Glyma.19G204200.1	CDS
19	46046257	0.763284	Landrace vs Wild	red	Glyma.19G204200.1	CDS
19	46048348	0.756091	Landrace vs Wild	red	Glyma.19G204200.1	CDS
19	46039187	0.754924	Landrace vs Wild	red	Glyma.19G204200.1	CDS
19	46046257	0.729741	Improved vs Wild	green	Glyma.19G204200.1	CDS
19	46048348	0.729603	Improved vs Wild	green	Glyma.19G204200.1	CDS
19	46086492	0.772242	Landrace vs Wild	red	Glyma.19G204700.1	CDS
19	46086492	0.743622	Improved vs Wild	green	Glyma.19G204700.1	CDS
19	46124056	0.637976	Landrace vs Wild	red	Glyma.19G205200.1	CDS
19	46124056	0.610482	Improved vs Wild	green	Glyma.19G205200.1	CDS
19	46124083	0.610326	Landrace vs Wild	red	Glyma.19G205200.1	CDS

Stable 3. SNPs and small InDels in the major QTL region

Chr	Position	Ref	Alt	Effect type	Gene name	Type	QTL
Chr06	11470193	T	TAATTCTG AAAACTGA	stop_gained	Glyma.06G140700	InDel	*qVGH6-1*
Chr06	11475127	ACC	A	frame_shief	Glyma.06G140600	InDel	*qVGH6-1*
Chr06	11475159	ACGACAT	A	codon_deletion	Glyma.06G140600	InDel	*qVGH6-1*
Chr06	11494867	T	TA	splice_site_region	Glyma.06G141100	InDel	*qVGH6-1*
Chr06	11435928	G	T	start_gained	Glyma.06G140100	SNP	*qVGH6-1*
Chr06	11437214	G	A	non_synonymous	Glyma.06G140100	SNP	*qVGH6-1*
Chr06	11439628	T	A	non_synonymous	Glyma.06G140200	SNP	*qVGH6-1*
Chr06	11441256	A	C	non_synonymous	Glyma.06G140200	SNP	*qVGH6-1*
Chr06	11441469	T	A	start_gained	Glyma.06G140200	SNP	*qVGH6-1*
Chr06	11443459	G	A	splice_site_region	Glyma.06G140300	SNP	*qVGH6-1*
Chr06	11444065	A	C	splice_site_region	Glyma.06G140300	SNP	*qVGH6-1*
Chr06	11449817	T	C	non_synonymous	Glyma.06G140400	SNP	*qVGH6-1*
Chr06	11470251	A	G	non_synonymous	Glyma.06G140700	SNP	*qVGH6-1*
Chr06	11474894	C	G	non_synonymous	Glyma.06G140600	SNP	*qVGH6-1*
Chr06	11475217	A	G	start_gained	Glyma.06G140600	SNP	*qVGH6-1*
Chr06	11496362	G	T	non_synonymous	Glyma.06G141100	SNP	*qVGH6-1*
Chr06	11497220	T	G	non_synonymous	Glyma.06G141100	SNP	*qVGH6-1*
Chr06	11497236	A	G	non_synonymous	Glyma.06G141100	SNP	*qVGH6-1*
Chr06	11503372	C	T	non_synonymous	Glyma.06G141200	SNP	*qVGH6-1*
Chr06	11503396	C	A	non_synonymous	Glyma.06G141200	SNP	*qVGH6-1*
Chr06	11522083	G	C	non_synonymous	Glyma.06G141300	SNP	*qVGH6-1*

Chr06	11522684	A	T	non_synonymous	Glyma.06G141300	SNP	*qVGH6-1*
Chr06	11522690	A	T	non_synonymous	Glyma.06G141300	SNP	*qVGH6-1*
Chr13	1706509	G	GTTC	codon_insertion	Glyma.13G005800	InDel	*qVGH6-1*
Chr13	1806789	G	GT	frame_shief	Glyma.13G006000	InDel	*qVGH13-1*
Chr13	1848057	AATG	A	codon_deletion	Glyma.13G006200	InDel	*qVGH13-1*
Chr13	1853678	G	GA	frame_shief	Glyma.13G006300	InDel	*qVGH13-1*
Chr13	1949294	T	TA	frame_shief	Glyma.13G006800	InDel	*qVGH13-1*
Chr13	1962693	TC	T	frame_shief	Glyma.13G007100	InDel	*qVGH13-1*
Chr13	2095329	CA	C	frame_shief	Glyma.13G007700	InDel	*qVGH13-1*
Chr13	2476569	GTA	G	frame_shief	Glyma.13G008700	InDel	*qVGH13-1*
Chr13	2655987	G	GT	splice_site_region	Glyma.13G009800	InDel	*qVGH13-1*
Chr13	2833169	T	TTA	frame_shief	Glyma.13G010300	InDel	*qVGH13-1*
Chr13	2839809	GC	G	frame_shief	Glyma.13G010400	InDel	*qVGH13-1*
Chr13	1640028	C	T	non_synonymous	Glyma.13G005700	SNP	*qVGH13-1*
Chr13	1640830	C	T	non_synonymous	Glyma.13G005700	SNP	*qVGH13-1*
Chr13	1844334	T	C	start_gained	Glyma.13G006200	SNP	*qVGH13-1*
Chr13	1844449	A	G	non_synonymous	Glyma.13G006200	SNP	*qVGH13-1*
Chr13	1852696	T	C	non_synonymous	Glyma.13G006300	SNP	*qVGH13-1*
Chr13	1852900	C	T	non_synonymous	Glyma.13G006300	SNP	*qVGH13-1*
Chr13	1859005	G	A	non_synonymous	Glyma.13G006200	SNP	*qVGH13-1*
Chr13	1859929	T	G	non_synonymous	Glyma.13G006200	SNP	*qVGH13-1*
Chr13	1859940	G	T	non_synonymous	Glyma.13G006200	SNP	*qVGH13-1*
Chr13	1860009	A	T	non_synonymous	Glyma.13G006200	SNP	*qVGH13-1*
Chr13	1860021	C	A	non_synonymous	Glyma.13G006200	SNP	*qVGH13-1*
Chr13	1860141	G	T	non_synonymous	Glyma.13G006200	SNP	*qVGH13-1*
Chr13	1860502	T	G	non_synonymous	Glyma.13G006200	SNP	*qVGH13-1*
Chr13	1860514	A	T	non_synonymous	Glyma.13G006200	SNP	*qVGH13-1*
Chr13	1864527	C	T	non_synonymous	Glyma.13G006400	SNP	*qVGH13-1*
Chr13	1864541	G	A	non_synonymous	Glyma.13G006400	SNP	*qVGH13-1*
Chr13	1864800	C	A	non_synonymous	Glyma.13G006400	SNP	*qVGH13-1*
Chr13	1864958	C	T	stop_gained	Glyma.13G006400	SNP	*qVGH13-1*
Chr13	1865564	G	A	non_synonymous	Glyma.13G006400	SNP	*qVGH13-1*
Chr13	1897370	C	T	non_synonymous	Glyma.13G006600	SNP	*qVGH13-1*
Chr13	1933143	C	T	non_synonymous	Glyma.13G006700	SNP	*qVGH13-1*
Chr13	1949302	T	C	non_synonymous	Glyma.13G006800	SNP	*qVGH13-1*
Chr13	1949496	A	C	non_synonymous	Glyma.13G006800	SNP	*qVGH13-1*
Chr13	1950648	C	T	non_synonymous	Glyma.13G006900	SNP	*qVGH13-1*
Chr13	1962522	T	C	non_synonymous	Glyma.13G007100	SNP	*qVGH13-1*
Chr13	1962828	G	A	non_synonymous	Glyma.13G007100	SNP	*qVGH13-1*
Chr13	1969875	T	G	splice_site_region	Glyma.13G007200	SNP	*qVGH13-1*
Chr13	1970035	C	T	splice_site_region	Glyma.13G007200	SNP	*qVGH13-1*
Chr13	2008921	G	A	non_synonymous	Glyma.13G007300	SNP	*qVGH13-1*
Chr13	2009843	G	A	stop_gained	Glyma.13G007400	SNP	*qVGH13-1*
Chr13	2012163	G	A	non_synonymous	Glyma.13G007400	SNP	*qVGH13-1*

Chr13	2014664	A	C	non_synonymous	Glyma.13G007500	SNP	*qVGH13-1*
Chr13	2015111	A	G	non_synonymous	Glyma.13G007500	SNP	*qVGH13-1*
Chr13	2029233	T	A	non_synonymous	Glyma.13G007400	SNP	*qVGH13-1*
Chr13	2091676	A	G	splice_site_region	Glyma.13G007700	SNP	*qVGH13-1*
Chr13	2092170	T	C	non_synonymous	Glyma.13G007700	SNP	*qVGH13-1*
Chr13	2092401	C	G	non_synonymous	Glyma.13G007700	SNP	*qVGH13-1*
Chr13	2092562	G	C	non_synonymous	Glyma.13G007700	SNP	*qVGH13-1*
Chr13	2092812	A	C	non_synonymous	Glyma.13G007700	SNP	*qVGH13-1*
Chr13	2093253	A	C	non_synonymous	Glyma.13G007700	SNP	*qVGH13-1*
Chr13	2093823	A	G	non_synonymous	Glyma.13G007700	SNP	*qVGH13-1*
Chr13	2093870	C	T	stop_gained	Glyma.13G007700	SNP	*qVGH13-1*
Chr13	2093985	G	A	non_synonymous	Glyma.13G007700	SNP	*qVGH13-1*
Chr13	2094008	G	A	non_synonymous	Glyma.13G007700	SNP	*qVGH13-1*
Chr13	2094496	C	T	non_synonymous	Glyma.13G007700	SNP	*qVGH13-1*
Chr13	2094515	T	A	non_synonymous	Glyma.13G007700	SNP	*qVGH13-1*
Chr13	2095020	C	T	non_synonymous	Glyma.13G007700	SNP	*qVGH13-1*
Chr13	2095265	A	G	non_synonymous	Glyma.13G007700	SNP	*qVGH13-1*
Chr13	2095313	C	T	stop_gained	Glyma.13G007700	SNP	*qVGH13-1*
Chr13	2095403	G	A	stop_gained	Glyma.13G007700	SNP	*qVGH13-1*
Chr13	2095495	T	C	non_synonymous	Glyma.13G007700	SNP	*qVGH13-1*
Chr13	2095779	C	A	non_synonymous	Glyma.13G007700	SNP	*qVGH13-1*
Chr13	2095810	A	G	non_synonymous	Glyma.13G007700	SNP	*qVGH13-1*
Chr13	2096148	A	T	non_synonymous	Glyma.13G007700	SNP	*qVGH13-1*
Chr13	2118853	G	A	non_synonymous	Glyma.13G007800	SNP	*qVGH13-1*
Chr13	2120526	A	T	non_synonymous	Glyma.13G007800	SNP	*qVGH13-1*
Chr13	2121368	G	A	non_synonymous	Glyma.13G007800	SNP	*qVGH13-1*
Chr13	2121376	T	A	non_synonymous	Glyma.13G007800	SNP	*qVGH13-1*
Chr13	2122454	T	A	non_synonymous	Glyma.13G007800	SNP	*qVGH13-1*
Chr13	2127998	G	A	non_synonymous	Glyma.13G007900	SNP	*qVGH13-1*
Chr13	2128700	A	T	non_synonymous	Glyma.13G007900	SNP	*qVGH13-1*
Chr13	2129983	T	G	non_synonymous	Glyma.13G007900	SNP	*qVGH13-1*
Chr13	2233279	C	T	non_synonymous	Glyma.13G008000	SNP	*qVGH13-1*
Chr13	2250427	G	A	non_synonymous	Glyma.13G008100	SNP	*qVGH13-1*
Chr13	2250550	C	T	splice_site_acceptor	Glyma.13G008100	SNP	*qVGH13-1*
Chr13	2250939	C	T	non_synonymous	Glyma.13G008100	SNP	*qVGH13-1*
Chr13	2346958	G	A	non_synonymous	Glyma.13G008200	SNP	*qVGH13-1*
Chr13	2399207	T	G	non_synonymous	Glyma.13G008300	SNP	*qVGH13-1*
Chr13	2412668	T	C	non_synonymous	Glyma.13G008400	SNP	*qVGH13-1*
Chr13	2412705	C	A	non_synonymous	Glyma.13G008400	SNP	*qVGH13-1*
Chr13	2412752	C	T	non_synonymous	Glyma.13G008400	SNP	*qVGH13-1*
Chr13	2413040	C	T	non_synonymous	Glyma.13G008400	SNP	*qVGH13-1*
Chr13	2413211	T	C	non_synonymous	Glyma.13G008400	SNP	*qVGH13-1*
Chr13	2413406	A	G	non_synonymous	Glyma.13G008400	SNP	*qVGH13-1*
Chr13	2413473	T	C	non_synonymous	Glyma.13G008400	SNP	*qVGH13-1*

Chr13	2438737	A	T	non_synonymous	Glyma.13G008500	SNP	*qVGH13-1*
Chr13	2471142	T	A	stop_gained	Glyma.13G008600	SNP	*qVGH13-1*
Chr13	2476472	T	G	non_synonymous	Glyma.13G008700	SNP	*qVGH13-1*
Chr13	2512032	C	T	non_synonymous	Glyma.13G008800	SNP	*qVGH13-1*
Chr13	2512173	C	T	non_synonymous	Glyma.13G008800	SNP	*qVGH13-1*
Chr13	2535289	G	T	non_synonymous	Glyma.13G009000	SNP	*qVGH13-1*
Chr13	2566263	C	T	non_synonymous	Glyma.13G009200	SNP	*qVGH13-1*
Chr13	2566651	C	T	non_synonymous	Glyma.13G009200	SNP	*qVGH13-1*
Chr13	2566693	C	A	non_synonymous	Glyma.13G009200	SNP	*qVGH13-1*
Chr13	2566704	A	T	non_synonymous	Glyma.13G009200	SNP	*qVGH13-1*
Chr13	2566914	C	A	splice_site_region	Glyma.13G009200	SNP	*qVGH13-1*
Chr13	2568003	C	T	stop_gained	Glyma.13G009200	SNP	*qVGH13-1*
Chr13	2568012	G	T	non_synonymous	Glyma.13G009200	SNP	*qVGH13-1*
Chr13	2618209	T	C	non_synonymous	Glyma.13G009400	SNP	*qVGH13-1*
Chr13	2618405	A	G	non_synonymous	Glyma.13G009400	SNP	*qVGH13-1*
Chr13	2618413	A	T	non_synonymous	Glyma.13G009400	SNP	*qVGH13-1*
Chr13	2625854	T	C	non_synonymous	Glyma.13G009500	SNP	*qVGH13-1*
Chr13	2626915	T	A	non_synonymous	Glyma.13G009500	SNP	*qVGH13-1*
Chr13	2627146	T	G	non_synonymous	Glyma.13G009500	SNP	*qVGH13-1*
Chr13	2628187	T	A	non_synonymous	Glyma.13G009500	SNP	*qVGH13-1*
Chr13	2628628	A	G	non_synonymous	Glyma.13G009500	SNP	*qVGH13-1*
Chr13	2628704	C	T	non_synonymous	Glyma.13G009500	SNP	*qVGH13-1*
Chr13	2628953	G	C	non_synonymous	Glyma.13G009500	SNP	*qVGH13-1*
Chr13	2629117	C	A	non_synonymous	Glyma.13G009500	SNP	*qVGH13-1*
Chr13	2641650	G	A	non_synonymous	Glyma.13G009700	SNP	*qVGH13-1*
Chr13	2656160	C	A	non_synonymous	Glyma.13G009800	SNP	*qVGH13-1*
Chr13	2833684	G	A	non_synonymous	Glyma.13G010300	SNP	*qVGH13-1*
Chr13	8436671	A	ATTCT	frame_shief	Glyma.13G029100	InDel	*qVGH13-2*
Chr13	9112675	CTTCT	C	frame_shief	Glyma.13G029800	InDel	*qVGH13-2*
Chr13	8626619	A	T	non_synonymous	Glyma.13G029400	SNP	*qVGH13-2*
Chr13	8627266	G	A	non_synonymous	Glyma.13G029400	SNP	*qVGH13-2*
Chr13	8627509	G	A	non_synonymous	Glyma.13G029400	SNP	*qVGH13-2*
Chr13	8628210	G	A	non_synonymous	Glyma.13G029400	SNP	*qVGH13-2*
Chr13	9036028	A	T	non_synonymous	Glyma.13G029700	SNP	*qVGH13-2*
Chr13	9111159	G	A	non_synonymous	Glyma.13G029800	SNP	*qVGH13-2*
Chr13	39979426	CTA	C	splice_site_region	Glyma.13G302800	InDel	*qVGH13-3*
Chr13	40007092	GTACATGGA	G	frame_shief	Glyma.13G303100	InDel	*qVGH13-3*
Chr13	40008552	ACAAGTATAT ATAGGATATG TTGTTTGATTT ATACCTG	A	splice_site_donor	Glyma.13G303100	InDel	*qVGH13-3*
Chr13	40155077	T	TGTG	codon_insertion	Glyma.13G304600	InDel	*qVGH13-3*
Chr13	39977464	A	G	non_synonymous	Glyma.13G302800	SNP	*qVGH13-3*
Chr13	39978523	C	A	splice_site_acceptor	Glyma.13G302800	SNP	*qVGH13-3*

Chr13	39979827	C	A	start_gained	Glyma.13G302800	SNP	*qVGH13-3*
Chr13	40007179	A	C	non_synonymous	Glyma.13G303100	SNP	*qVGH13-3*
Chr13	40007455	C	A	non_synonymous	Glyma.13G303100	SNP	*qVGH13-3*
Chr13	40008634	G	A	non_synonymous	Glyma.13G303100	SNP	*qVGH13-3*
Chr13	40008648	C	G	non_synonymous	Glyma.13G303100	SNP	*qVGH13-3*
Chr13	40048948	A	T	non_synonymous	Glyma.13G303400	SNP	*qVGH13-3*
Chr13	40053423	A	C	non_synonymous	Glyma.13G303500	SNP	*qVGH13-3*
Chr13	40091120	A	G	non_synonymous	Glyma.13G303800	SNP	*qVGH13-3*
Chr13	40091547	G	A	non_synonymous	Glyma.13G303800	SNP	*qVGH13-3*
Chr13	40091637	T	A	non_synonymous	Glyma.13G303800	SNP	*qVGH13-3*
Chr13	40091969	C	G	non_synonymous	Glyma.13G303800	SNP	*qVGH13-3*
Chr13	40091975	G	T	non_synonymous	Glyma.13G303800	SNP	*qVGH13-3*
Chr13	40092128	T	A	non_synonymous	Glyma.13G303800	SNP	*qVGH13-3*
Chr13	40092373	G	C	start_gained	Glyma.13G303800	SNP	*qVGH13-3*
Chr13	40099952	A	C	non_synonymous	Glyma.13G303900	SNP	*qVGH13-3*
Chr13	40117293	G	C	non_synonymous	Glyma.13G304000	SNP	*qVGH13-3*
Chr13	40119742	T	C	non_synonymous	Glyma.13G304100	SNP	*qVGH13-3*
Chr13	40141801	C	T	non_synonymous	Glyma.13G304500	SNP	*qVGH13-3*
Chr13	40156334	G	A	stop_gained	Glyma.13G304600	SNP	*qVGH13-3*
Chr13	40156795	C	T	non_synonymous	Glyma.13G304600	SNP	*qVGH13-3*
Chr13	40160506	T	C	splice_site_donor	Glyma.13G304700	SNP	*qVGH13-3*
Chr13	40161491	A	C	STOP_LOST	Glyma.13G304700	SNP	*qVGH13-3*
Chr13	40161837	T	C	non_synonymous	Glyma.13G304800	SNP	*qVGH13-3*
Chr13	40161862	A	G	non_synonymous	Glyma.13G304800	SNP	*qVGH13-3*
Chr13	40161898	G	C	non_synonymous	Glyma.13G304800	SNP	*qVGH13-3*
Chr13	40162007	G	A	non_synonymous	Glyma.13G304800	SNP	*qVGH13-3*
Chr13	40176835	G	A	non_synonymous	Glyma.13G305000	SNP	*qVGH13-3*
Chr13	40180722	G	A	non_synonymous	Glyma.13G305000	SNP	*qVGH13-3*
Chr13	40180821	G	A	non_synonymous	Glyma.13G305000	SNP	*qVGH13-3*
Chr19	45043376	ACT	A	splice_site_region	Glyma.19G192700	InDel	*qVGH19-1.1*
Chr19	45934239	T	TC	frame_shief	Glyma.19G202400	InDel	*qVGH19-1.2*
Chr19	45934483	G	GTT	splice_site_region	Glyma.19G202400	InDel	*qVGH19-1.2*
Chr19	45934533	A	AGT	frame_shief	Glyma.19G202400	InDel	*qVGH19-1.2*
Chr19	45950057	TA	T	frame_shief	Glyma.19G202800	InDel	*qVGH19-1.2*
Chr19	45950073	A	AT	frame_shief	Glyma.19G202800	InDel	*qVGH19-1.2*
Chr19	45071789	G	T	non_synonymous	Glyma.19G192900	SNP	*qVGH19-1.1*
Chr19	45075111	T	C	non_synonymous	Glyma.19G192900	SNP	*qVGH19-1.1*
Chr19	45077823	G	A	non_synonymous	Glyma.19G192900	SNP	*qVGH19-1.1*
Chr19	45077858	T	C	start_gained	Glyma.19G192900	SNP	*qVGH19-1.1*
Chr19	45087020	T	A	non_synonymous	Glyma.19G193100	SNP	*qVGH19-1.1*
Chr19	45087102	A	C	non_synonymous	Glyma.19G193100	SNP	*qVGH19-1.1*
Chr19	45087110	T	C	non_synonymous	Glyma.19G193100	SNP	*qVGH19-1.1*
Chr19	45087390	C	A	non_synonymous	Glyma.19G193100	SNP	*qVGH19-1.1*
Chr19	45089587	A	C	splice_site_region	Glyma.19G193100	SNP	*qVGH19-1.1*

Chr19	45114951	A	G	non_synonymous	Glyma.19G193400	SNP	*qVGH19-1.1*
Chr19	45164521	A	T	non_synonymous	Glyma.19G194100	SNP	*qVGH19-1.1*
Chr19	45184804	C	A	non_synonymous	Glyma.19G194300	SNP	*qVGH19-1.1*
Chr19	45200508	A	T	non_synonymous	Glyma.19G194400	SNP	*qVGH19-1.1*
Chr19	45203982	G	A	non_synonymous	Glyma.19G194500	SNP	*qVGH19-1.1*
Chr19	45204441	A	C	non_synonymous	Glyma.19G194500	SNP	*qVGH19-1.1*
Chr19	45208309	T	A	non_synonymous	Glyma.19G194600	SNP	*qVGH19-1.1*
Chr19	45209242	A	G	splice_site_region	Glyma.19G194600	SNP	*qVGH19-1.1*
Chr19	45923578	T	G	non_synonymous	Glyma.19G202300	SNP	*qVGH19-1.2*
Chr19	45933900	C	T	non_synonymous	Glyma.19G202400	SNP	*qVGH19-1.2*
Chr19	45933957	A	T	stop_gained	Glyma.19G202400	SNP	*qVGH19-1.2*
Chr19	45933964	C	T	non_synonymous	Glyma.19G202400	SNP	*qVGH19-1.2*
Chr19	45933986	G	T	non_synonymous	Glyma.19G202400	SNP	*qVGH19-1.2*
Chr19	45934021	C	T	non_synonymous	Glyma.19G202400	SNP	*qVGH19-1.2*
Chr19	45934048	C	T	non_synonymous	Glyma.19G202400	SNP	*qVGH19-1.2*
Chr19	45934147	G	A	splice_site_region	Glyma.19G202400	SNP	*qVGH19-1.2*
Chr19	45934185	G	A	stop_gained	Glyma.19G202400	SNP	*qVGH19-1.2*
Chr19	45934509	G	A	non_synonymous	Glyma.19G202400	SNP	*qVGH19-1.2*
Chr19	45950170	G	A	non_synonymous	Glyma.19G202800	SNP	*qVGH19-1.2*
Chr19	45950265	C	G	non_synonymous	Glyma.19G202800	SNP	*qVGH19-1.2*
Chr19	45950401	A	G	non_synonymous	Glyma.19G202800	SNP	*qVGH19-1.2*
Chr19	45990686	C	A	non_synonymous	Glyma.19G203200	SNP	*qVGH19-1.2*
Chr19	45991024	A	G	non_synonymous	Glyma.19G203200	SNP	*qVGH19-1.2*
Chr19	45999678	C	G	non_synonymous	Glyma.19G203400	SNP	*qVGH19-1.2*
Chr19	45999764	A	C	non_synonymous	Glyma.19G203400	SNP	*qVGH19-1.2*
Chr19	45999775	T	A	non_synonymous	Glyma.19G203400	SNP	*qVGH19-1.2*
Chr19	45999915	G	A	non_synonymous	Glyma.19G203400	SNP	*qVGH19-1.2*
Chr19	46000120	T	G	non_synonymous	Glyma.19G203400	SNP	*qVGH19-1.2*
Chr19	46000435	T	C	non_synonymous	Glyma.19G203400	SNP	*qVGH19-1.2*
Chr19	46000505	G	T	non_synonymous	Glyma.19G203400	SNP	*qVGH19-1.2*
Chr19	46001553	A	T	non_synonymous	Glyma.19G203400	SNP	*qVGH19-1.2*
Chr19	46002449	G	T	non_synonymous	Glyma.19G203400	SNP	*qVGH19-1.2*
Chr19	46002989	A	T	non_synonymous	Glyma.19G203400	SNP	*qVGH19-1.2*
Chr19	46003236	C	T	non_synonymous	Glyma.19G203400	SNP	*qVGH19-1.2*
Chr19	46003251	C	T	non_synonymous	Glyma.19G203400	SNP	*qVGH19-1.2*
Chr19	46003289	G	T	non_synonymous	Glyma.19G203400	SNP	*qVGH19-1.2*
Chr19	46003999	T	C	splice_site_region	Glyma.19G203400	SNP	*qVGH19-1.2*
Chr19	46004140	G	A	non_synonymous	Glyma.19G203400	SNP	*qVGH19-1.2*
Chr19	46004174	T	G	non_synonymous	Glyma.19G203400	SNP	*qVGH19-1.2*
Chr19	46004404	T	C	non_synonymous	Glyma.19G203400	SNP	*qVGH19-1.2*
Chr19	46007619	C	T	non_synonymous	Glyma.19G203500	SNP	*qVGH19-1.2*
Chr19	46009432	T	A	non_synonymous	Glyma.19G203600	SNP	*qVGH19-1.2*
Chr19	46009487	G	A	non_synonymous	Glyma.19G203600	SNP	*qVGH19-1.2*
Chr19	46016046	C	T	non_synonymous	Glyma.19G203800	SNP	*qVGH19-1.2*

Chr19	46019017	C	T	non_synonymous	Glyma.19G203900	SNP	*qVGH19-1.2*
Chr19	46019643	A	G	non_synonymous	Glyma.19G203900	SNP	*qVGH19-1.2*
Chr19	46020697	T	A	start_gained	Glyma.19G203900	SNP	*qVGH19-1.2*
Chr19	46057306	G	A	non_synonymous	Glyma.19G204300	SNP	*qVGH19-1.2*
Chr19	46073299	A	C	non_synonymous	Glyma.19G204500	SNP	*qVGH19-1.2*
Chr19	46080911	T	G	non_synonymous	Glyma.19G204600	SNP	*qVGH19-1.2*

Stable 4 The FPKM of different tissue sample for candidate genes in major QTL region

gene_id	pod	root_hairs	leaves	root	nodules	seed	sam	stem	flower
Glyma.06G140000	4.97	7.68	3.16	6.30	4.97	1.22	13.96	1.96	1.87
Glyma.06G140100	0.11	0.37	0.02	0.43	0.54	0.09	0.17	0.65	1.21
Glyma.06G140200	8.86	3.33	12.93	2.29	3.77	5.21	10.27	9.53	9.43
Glyma.06G140300	8.33	6.53	5.72	6.06	6.97	10.69	5.53	5.69	4.59
Glyma.06G140400	9.68	7.43	8.24	9.60	10.59	8.67	6.97	9.39	16.26
Glyma.06G140500	0.06	0.31	0.11	0.07	0.00	0.00	2.06	0.38	0.50
Glyma.06G140600	10.99	8.10	14.61	10.11	8.64	9.59	10.02	9.35	13.49
Glyma.06G140700	0.33	0.19	0.43	0.15	0.11	0.44	0.13	0.05	0.26
Glyma.06G140800	0.15	0.10	0.19	0.00	0.09	0.39	0.03	0.12	0.00
Glyma.06G140900	26.70	72.46	18.73	73.14	50.96	107.92	44.55	40.22	22.47
Glyma.06G141000	10.65	11.46	8.73	9.24	8.47	20.56	6.35	7.24	5.02
Glyma.06G141100	19.61	12.80	4.93	4.88	15.65	11.09	19.87	32.24	4.74
Glyma.06G141200	4.61	17.53	0.78	10.30	6.40	11.21	8.78	12.97	1.47
Glyma.06G141300	5.60	11.64	1.91	13.10	9.44	5.62	8.27	8.66	3.67
Glyma.13G005700	0.00	0.01	0.00	0.00	0.00	0.00	0.00	0.02	0.00
Glyma.13G005800	1.72	1.73	0.61	1.26	1.70	0.57	5.01	1.37	0.26
Glyma.13G005900	0.00	0.00	0.00	0.00	0.00	0.00	0.00	0.00	0.00
Glyma.13G006000	0.04	0.00	0.00	0.00	0.00	0.41	0.16	0.00	0.00
Glyma.13G006100	0.00	0.00	0.00	0.00	0.00	0.00	0.00	0.00	0.00
Glyma.13G006200	2.87	1.52	3.44	1.65	2.82	4.04	2.87	2.24	2.95
Glyma.13G006300	0.03	0.00	0.00	0.00	0.11	0.17	0.00	0.00	0.00
Glyma.13G006400	0.00	0.00	0.03	0.00	0.00	0.00	0.00	0.00	0.00
Glyma.13G006500	0.42	1.58	0.07	2.13	2.69	0.53	0.87	3.02	0.19
Glyma.13G006600	0.00	0.05	0.05	0.00	0.00	0.05	0.45	0.05	0.00
Glyma.13G006700	0.03	0.25	6.20	1.89	0.65	0.00	0.00	0.14	0.20
Glyma.13G006800	0.48	0.08	0.10	0.00	0.00	1.00	0.09	0.00	0.58
Glyma.13G006900	0.24	0.00	0.11	0.00	0.00	1.11	0.00	0.11	0.14
Glyma.13G007000	1.67	0.49	3.91	2.67	0.69	0.51	1.47	2.35	4.94
Glyma.13G007100	0.13	0.00	0.53	0.97	0.47	0.00	0.24	0.46	1.33
Glyma.13G007200	6.29	1.22	36.65	2.36	1.68	2.39	3.76	6.05	31.67
Glyma.13G007300	0.53	0.39	0.00	0.00	0.00	0.00	0.00	0.00	0.00
Glyma.13G007400	1.15	1.70	1.72	1.48	1.78	2.51	1.66	1.19	1.08
Glyma.13G007500	0.00	0.00	0.00	0.00	0.00	0.06	0.00	0.00	0.00
Glyma.13G007600	0.00	0.00	0.00	0.00	0.00	0.00	0.00	0.00	0.00

Glyma.13G007700	0.00	0.00	0.00	0.00	0.00	0.00	0.00	0.00	0.00
Glyma.13G007800	1.79	1.27	1.54	0.67	0.92	2.28	2.60	1.38	0.94
Glyma.13G007900	0.09	0.08	0.02	0.03	0.02	0.02	0.07	0.04	0.04
Glyma.13G008000	7.35	5.35	3.16	3.92	6.77	8.33	15.56	12.25	3.04
Glyma.13G008100	0.00	0.00	0.00	0.00	0.00	0.00	0.00	0.13	0.00
Glyma.13G008200	11.60	14.61	12.32	11.54	13.12	8.46	12.42	10.66	10.73
Glyma.13G008300	3.92	3.46	5.06	3.04	4.01	3.62	3.74	3.90	2.49
Glyma.13G008400	0.00	0.00	0.00	0.00	0.00	0.00	0.00	0.00	0.00
Glyma.13G008500	1.91	3.54	0.24	1.10	2.84	2.71	3.86	1.35	0.85
Glyma.13G008600	0.17	0.11	0.00	0.06	0.00	0.36	0.19	0.03	0.14
Glyma.13G008700	0.05	0.18	0.29	0.05	0.05	0.00	1.02	0.15	0.17
Glyma.13G008800	0.00	0.00	0.00	0.00	0.00	0.00	0.03	0.00	0.04
Glyma.13G008900	0.95	0.16	0.40	0.31	0.00	0.04	4.01	1.09	1.16
Glyma.13G009000	0.03	0.43	0.00	0.96	0.13	0.10	0.00	0.04	0.09
Glyma.13G009100	14.48	12.29	18.16	12.68	5.27	36.39	19.68	14.06	38.02
Glyma.13G009200	0.00	0.00	0.00	0.00	0.00	0.00	0.00	0.00	0.00
Glyma.13G009300	0.53	0.82	12.68	3.39	1.24	2.20	2.42	1.82	6.58
Glyma.13G009400	0.00	0.00	0.00	0.00	0.00	0.00	0.00	0.00	0.00
Glyma.13G009500	0.80	0.72	0.72	1.23	2.44	0.66	0.86	0.70	0.97
Glyma.13G009600	0.08	0.13	0.18	0.09	0.23	0.13	0.15	0.22	0.37
Glyma.13G009700	0.44	0.88	0.05	0.55	0.83	0.41	3.32	0.50	0.27
Glyma.13G009800	13.13	14.21	18.50	11.79	10.78	15.13	20.46	15.93	18.79
Glyma.13G009900	0.62	1.68	0.02	0.60	0.87	0.38	3.05	1.34	0.36
Glyma.13G010000	0.56	0.02	0.09	0.00	0.00	0.06	0.28	0.09	0.05
Glyma.13G010100	17.23	9.38	7.12	13.33	12.93	36.95	9.80	10.18	8.97
Glyma.13G010200	7.18	5.90	25.84	6.79	6.85	3.73	11.73	5.78	10.70
Glyma.13G010300	0.00	0.00	0.00	0.00	0.00	0.00	0.00	0.00	0.00
Glyma.13G010400	1.09	0.00	0.00	0.00	0.00	0.00	0.00	0.00	0.00
Glyma.13G010500	7.89	4.00	24.60	3.01	2.23	12.47	11.48	6.69	6.04
Glyma.13G010600	0.00	0.00	0.00	0.00	0.00	0.00	0.00	0.00	0.00
Glyma.13G010700	0.97	2.02	0.43	0.89	1.93	1.50	2.92	0.85	0.88
Glyma.13G010800	0.00	0.00	0.00	0.03	0.00	0.00	0.00	0.01	0.00
Glyma.13G010900	0.15	0.05	0.11	0.10	0.06	0.25	0.64	0.08	0.38
Glyma.13G029500	0.30	9.56	21.13	1.11	38.84	0.07	3.40	9.08	2.18
Glyma.13G029600	1.13	0.38	3.02	0.87	0.64	1.36	0.74	1.81	0.67
Glyma.13G029700	3.75	2.74	3.49	2.02	2.51	2.45	2.51	3.13	3.01
Glyma.13G029800	0.00	0.00	0.00	0.00	0.00	0.01	0.00	0.00	0.00
Glyma.13G302800	1.47	6.48	12.77	13.75	7.68	0.69	2.40	6.12	14.05
Glyma.13G302900	72.37	8.86	269.47	9.06	6.44	37.23	94.77	65.10	31.79
Glyma.13G303000	4.30	2.96	0.21	1.80	1.70	3.81	11.83	2.36	1.25
Glyma.13G303100	0.00	0.00	0.00	0.00	0.00	0.00	0.00	0.00	0.00
Glyma.13G303200	0.00	0.03	0.00	0.00	0.00	0.00	0.00	0.00	0.13
Glyma.13G303300	0.10	0.00	0.55	0.00	0.00	0.00	0.00	0.02	0.16
Glyma.13G303400	0.08	0.42	1.35	3.12	0.66	0.16	0.79	0.24	1.30

Glyma.13G303500	1.36	1.61	7.44	0.89	1.01	1.09	3.06	1.39	3.20
Glyma.13G303600	16.92	26.11	6.14	42.37	32.10	14.31	15.16	18.01	47.40
Glyma.13G303700	5.19	11.25	20.42	30.11	10.64	1.17	4.53	5.35	13.35
Glyma.13G303700	5.19	11.25	20.42	30.11	10.64	1.17	4.53	5.35	13.35
Glyma.13G303800	1.04	0.18	11.99	0.06	0.47	0.00	0.58	2.25	3.35
Glyma.13G303900	0.00	1.53	0.19	3.85	2.63	0.00	0.00	0.03	0.07
Glyma.13G304000	0.01	0.62	0.16	4.50	0.28	0.02	0.11	0.53	1.45
Glyma.13G304100	1.41	1.19	0.50	0.10	3.57	11.57	0.20	0.96	0.43
Glyma.13G304200	0.89	0.03	1.22	0.45	0.03	0.61	3.24	1.54	1.08
Glyma.13G304300	14.14	15.86	7.04	24.18	18.94	25.26	25.44	11.82	11.72
Glyma.13G304400	15.98	57.59	1.31	57.74	29.33	11.02	54.73	33.13	8.66
Glyma.13G304500	0.45	0.04	5.06	1.12	0.00	0.00	13.05	0.32	0.60
Glyma.13G304600	0.00	0.00	0.00	0.00	0.41	0.00	0.00	0.00	0.00
Glyma.13G304700	0.12	0.09	0.00	0.14	0.60	0.16	0.04	0.11	0.00
Glyma.13G304800	0.06	0.16	0.00	0.37	0.35	0.48	0.00	0.04	0.00
Glyma.13G304900	0.99	0.21	0.03	0.27	0.10	9.19	6.33	0.09	0.00
Glyma.13G305000	7.54	5.12	8.74	3.72	6.45	8.83	7.15	6.40	11.65
Glyma.19G192600	30.50	11.22	33.11	25.86	18.36	7.99	20.93	31.23	33.04
Glyma.19G192700	0.72	0.55	0.00	0.81	0.48	1.00	5.74	0.39	3.39
Glyma.19G192800	23.80	11.94	36.82	2.21	4.20	26.94	14.72	73.98	6.87
Glyma.19G192900	0.02	7.24	0.00	11.49	17.98	0.16	0.04	0.44	0.04
Glyma.19G193000	0.00	0.00	0.00	0.00	0.00	0.00	0.00	0.00	0.31
Glyma.19G193100	3.06	2.42	0.70	1.77	3.18	3.49	4.32	2.58	1.99
Glyma.19G193200	29.84	85.21	35.49	85.76	65.13	81.52	78.93	53.42	23.32
Glyma.19G193300	3.97	0.54	9.23	0.97	0.49	0.47	2.82	15.30	2.06
Glyma.19G193400	0.88	1.56	0.71	1.23	0.31	2.84	1.37	0.41	0.29
Glyma.19G193500	8.07	8.97	1.12	2.45	6.78	14.52	11.12	16.65	1.83
Glyma.19G193600	3.36	0.82	0.31	3.60	3.22	0.06	1.88	7.34	0.35
Glyma.19G193700	5.92	38.91	7.01	81.06	48.78	2.23	6.37	3.91	11.97
Glyma.19G193800	36.90	38.80	81.57	65.68	42.76	30.95	26.18	46.03	88.66
Glyma.19G193900	3.27	0.80	0.78	0.52	1.05	2.80	0.22	1.81	37.31
Glyma.19G194000	1.76	2.29	0.20	0.55	0.90	17.35	0.83	1.04	11.66
Glyma.19G194100	1.27	0.69	0.00	0.52	0.70	1.74	1.61	0.71	0.17
Glyma.19G194200	6.93	9.91	7.24	5.70	5.11	7.22	4.46	8.32	7.43
Glyma.19G194300	0.00	2.76	0.00	0.45	1.34	0.04	1.82	0.10	0.00
Glyma.19G194400	0.52	0.13	1.03	0.06	0.03	1.07	0.18	0.18	0.04
Glyma.19G194500	0.23	0.91	0.03	0.37	0.31	4.95	0.42	1.35	0.27
Glyma.19G194600	2.09	2.84	2.19	3.62	2.67	2.72	2.63	2.56	2.82
Glyma.19G202300	0.02	0.32	4.84	0.43	0.15	0.00	0.07	0.00	0.05
Glyma.19G202400	4.95	7.63	2.12	5.16	8.31	5.24	11.86	5.12	2.86
Glyma.19G202500	0.33	0.24	0.48	0.16	0.53	0.23	0.26	0.33	0.16
Glyma.19G202600	5.12	5.43	3.17	4.27	5.68	6.94	4.76	4.35	1.35
Glyma.19G202700	0.00	0.00	0.00	0.53	0.07	0.00	0.00	0.00	0.13
Glyma.19G202800	0.42	0.25	0.14	0.44	0.35	0.46	0.58	0.88	5.88

Glyma.19G202900	0.07	4.17	29.58	4.57	5.26	0.04	0.10	0.56	2.93
Glyma.19G203000	1.95	1.64	0.24	1.70	1.66	3.13	0.97	0.79	1.03
Glyma.19G203100	4.05	2.19	3.22	3.67	3.22	7.01	3.65	3.50	2.14
Glyma.19G203200	10.53	6.93	5.94	8.92	6.11	10.51	5.81	10.72	10.45
Glyma.19G203300	110.61	287.37	159.57	233.05	212.45	319.46	205.83	248.26	141.14
Glyma.19G203400	0.14	0.64	0.27	0.10	0.86	0.20	0.63	2.97	0.08
Glyma.19G203500	1.42	4.23	0.02	2.76	3.61	1.52	6.02	1.91	0.74
Glyma.19G203600	0.21	0.23	0.23	0.38	0.20	0.27	0.28	0.58	0.29
Glyma.19G203700	0.01	0.15	0.23	3.16	0.30	0.00	0.01	0.01	0.00
Glyma.19G203800	0.00	0.09	0.17	0.03	0.14	0.00	0.00	0.00	0.00
Glyma.19G203900	1.14	0.55	7.28	2.26	1.12	0.00	2.54	1.34	2.49
Glyma.19G204000	10.63	9.04	8.40	9.19	9.21	13.38	7.32	10.94	10.53
Glyma.19G204100	6.13	3.45	2.74	6.01	4.71	4.39	3.62	5.84	3.59
Glyma.19G204200	3.94	3.83	3.04	3.53	4.35	4.66	3.18	3.04	3.85
Glyma.19G204300	0.00	0.00	0.00	0.00	0.00	0.00	0.00	0.00	0.00
Glyma.19G204400	29.01	31.30	23.45	24.07	22.22	36.88	27.06	33.53	15.92
Glyma.19G204500	11.92	13.90	20.20	13.54	14.58	8.75	10.44	14.85	23.67
Glyma.19G204600	2.30	1.77	0.18	0.57	0.71	5.58	8.23	1.46	1.07
Glyma.19G204700	3.79	2.47	3.36	1.62	1.96	2.53	2.17	2.58	1.74
Glyma.19G204800	15.54	22.02	17.93	14.67	18.58	22.90	16.34	17.44	14.91
Glyma.19G204900	0.12	0.43	0.00	0.22	0.56	0.05	0.34	1.21	0.04
Glyma.19G205000	119.47	284.96	104.14	178.26	201.57	650.50	159.51	129.81	55.13
